# Job Satisfaction and Its Determinants among Nurse Anesthetists in Clinical Practice: The Botswana Experience

**DOI:** 10.1155/2021/5739584

**Published:** 2021-09-07

**Authors:** Mamo Woldu Kassa, Alemayehu Ginbo Bedada

**Affiliations:** ^1^Department of Anesthesia and Critical Care, Faculty of Medicine, University of Botswana, Gaborone, Botswana; ^2^Department of Surgery, Faculty of Medicine, University of Botswana, Gaborone, Botswana

## Abstract

Job satisfaction (JS) correlates positively with patients' satisfaction and outcomes and employees' well-being. In Botswana, the level of job satisfaction and its determinants among nurse anesthetists were not investigated. A cross-sectional study was conducted from January 2020 to June 2020 encompassing all nurse anesthetists in clinical practice in Botswana. A self-administered questionnaire was used that incorporated demographic data, reasons to stay on or leave their job, and a validated 20-item short form of the Minnesota Satisfaction Questionnaire which was pretested on five of our nurse anesthetists. Percentage is used to describe the data. The independence of categorical variables was examined using chi-square or Fisher's exact test. *p* value <0.05 was considered statistically significant. In Botswana, a total of 76 nurse anesthetists were in clinical practice during the study period. Sixty-six (86.9%) responded to the survey. Gender distribution was even, 50.0%. The overall JS was 36.4%. Males had significantly higher JS than females, *p* = 0.001. Significantly higher job satisfaction was found in married nurse anesthetists (*p* = 0.039), expatriate nurse anesthetists (*p* = 0.001), nurse anesthetists in non-referral hospitals (*p* = 0.023), and nurse anesthetists with ≥10 years' experience (*p* = 0.019). Nurse anesthetists were satisfied with security, social service, authority, ability utilization, and responsibility in ≥60.0% of the cases. They were not satisfied in compensation, working condition, and advancement in a similar percentage. The main reason to stay on their job was to serve the public in 68.2%. In Botswana, employers should make an effort to address the working conditions, compensation, and advancement of nurse anesthetists in clinical practice.

## 1. Introduction

The effectiveness of a functional healthcare delivery system depends to a large degree on human resource utilization and motivation [1, 2]. Out of the seven billion people globally, about five billion people do not have access to safe and affordable surgical and anesthesia care, and this is disproportionally high in low- and middle-income countries (LMICs) [3]. Job satisfaction (JS) is a balance outcome in an individual who expects fulfilment from his/her job and what he/she actually felt achieved [4–7]. Job satisfaction is an important indicator of the performance and efficiency of the healthcare system [1, 8]. Anesthetists play a pivotal role in a well-functioning surgical care system [4, 5, 9]. In most LMICs, the shortage of anesthesia care providers limited the access to safe anesthesia services [10–12]. Nurse anesthetists work under direct or indirect supervision of physician anesthesiologists in high-income countries, but in many LMICs, anesthesia is administered primarily by nurse anesthetists [13, 14]. A tense operation theatre environment and the need for teamwork predispose nurse anesthetists to a higher degree of stress that influences their professional and personal life [1, 6, 15–18]. The practice of anesthesia profession requires a smooth cooperation among different disciplines that are expected to perform with the highest safety standards and free of adverse events; this has tremendous impact on clinicians' attitudes towards their work and practice [15, 19, 20].

Factors that improve the level of JS include job rotations, senior teaching or support to cope with difficult situations, non-salary incentives, job recognition, professional development, and a great sense of organizational justice [6, 9, 13, 21]. Leaders who engage and promote effective bidirectional communication, foster trust, help employees to maintain a reasonable work-life balance, and flatten the hierarchy are successful in enhancing the level of JS of their co-workers [6]. Satisfied employees had a higher score in patients' satisfaction surveys [4, 6, 22]. Job satisfaction correlates positively with employee well-being and patient's satisfaction and outcome [2, 7, 16, 22, 23].

Job satisfaction which is based only on financial incentive is often temporary [6]. Evaluating employees' JS regularly to improve their work environment helps to develop strategies and coping mechanisms to address professional stress [6, 21]. Factors that affect JS are different in different countries and vary from time to time [24]. In view of the stressful working conditions with consequential high turnover, absenteeism, tardiness, waste, grievances, and accidents [4, 5, 15, 22, 24, 25], we designed this study to assess the JS and characterize its determinants among nurse anesthetists in clinical practice in Botswana with a goal of informing our findings to health policy makers and managers.

## 2. Methods and Materials

### 2.1. Study Setting

Botswana has a total population of about 2.3 million. The public health system serves about 85% of the population, while the remaining 15% subscribe to a private health sector. There are a total of 19 general (referral and district) hospitals and 17 primary hospitals in the country [26]. Currently, there are 76 nurse anesthetists practicing in Botswana. Nurse anesthetists provide anesthesia services at general and primary hospitals in Botswana, and they are the sole anesthesia providers in all primary hospitals [14]. To ensure confidentiality, no individual identifiers were used during data collection and analysis. The University of Botswana and Ministry of Health and Wellness of Botswana Institutional Review Boards granted permission to undertake this study.

### 2.2. Study Design

This is a cross-sectional study involving all nurse anesthetists in clinical practice in Botswana. It was conducted from January 2020 to June 2020.

### 2.3. Study Participants

All nurse anesthetists in clinical practice in Botswana at all levels of public and private hospitals were invited to participate in this study.

### 2.4. Sample Size and Sampling Technique

All the 76 nurse anesthetists currently in clinical practice in Botswana were invited to participate in this study. All nurse anesthetists who consented to participate are included in the survey.

### 2.5. Data Collection

Job satisfaction was assessed using self-administered questionnaire sent to all nurse anesthetists through a one-to-one e-mail or using the new end-to-end encrypted WhatsApp platform that blocks interception by a third party. The self-administered questionnaire has two parts. Part I contains basic demographic data (age, gender, citizenship, working hours per week, marital status, and level of education), health sector (public or private), and reasons to stay or leave the profession ( job dissatisfaction,job satisfaction, family related, personal reasons, change of profession, further education, and serving the public). The overall satisfaction level was recorded on a separate scale as satisfied or not satisfied. Part II contains the validated English version of the 20-item short form of Minnesota Satisfaction Questionnaire (MSQ) which is based on 5-point Likert-type scale that ranges from 1 point, very dissatisfied, to 5 points, very satisfied. The clarity of the questionnaire was checked on five nurse anesthetists, and no modification was required. All 76 potential participant nurse anesthetists were informed about the objectives and the details of the study by the principal investigator. Nurse anesthetists who agreed to participate in this study gave written consent.

### 2.6. Data Analysis

The data were checked for completeness and entered into an Excel spreadsheet which was transported to IBM Statistical Package for Social Sciences Statistics 27 for analysis. Very dissatisfied and dissatisfied responses in MSQ were considered as not satisfied, while satisfied and very satisfied responses were considered as satisfied. Percentage was used to describe the data. Categorical variables were compared using chi-square or Fisher's exact test. Two-tailed *p* values <0.05 were considered statistically significant.

## 3. Results

### 3.1. Demographics

**Figure 1:**
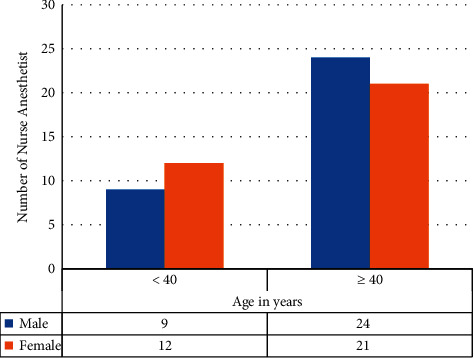
Gender and age distribution of nurse anesthetists who participated in the survey, Botswana, 2020.

In Botswana, a total of 76 nurse anesthetists were in clinical practice during the study period. Sixty-six of them, 86.9%, responded to the survey. Nurse anesthetists in the age group 40 years and older constituted 68.2%. The number of male and female nurse anesthetists who responded to the questionnaire was equal, 50.0% each (Figure 1).

The majority of nurse anesthetists were locals (Batswana) (71.2%); among the expatriates, 19.7% were Zimbabweans, 4.5% were Zambians, 3.0% were Malawians, and 1.5% were Tanzanians. The majority of Batswana nurse anesthetists were female (63.8%), while most of the expatriate nurse anesthetists were males (82.4%). Gender distribution among age groups, education levels, hospital types, relation with surgeons, working hours per week, years of experience, and senior backups was not significantly different. The marriage rate was 66.7%. Significantly more males were married than females, 81.8% vs. 53.1%, *p*=0.013. A majority, 92.4%, of our nurse anesthetists had diploma credentials; the remaining had a degree in anesthesia. The overall job satisfaction of the nurse anesthetists in our study was 36.4%. The JS of males was significantly higher than that of females, 57.6% vs. 15.2%, *p*=0.001. Statistically significant JS was found in nurse anesthetists who are married, 45.5% vs. 19.0%, *p*=0.039, expatriate nurse anesthetists, 68.4% vs. 23.4%, *p*=0.001, nurse anesthetists working in non-referral hospitals, 43.4% vs. 7.7%, *p*=0.023, and those nurse anesthetists with 10 years and more work experience, 46.5% vs. 17.4%, *p*=0.019 (Table 1). Job satisfaction was similar among the two age groups (<40 years and ≥40 years), *p*=0.362, educational levels (diploma or degree), *p*=0.645, health sector (public or private), *p*=0.103, relationship with surgeons (good or poor), *p*=1.000, the number of working hours per week (<40 hours or ≥ 40 hours), *p*=0.426, and senior staff backup (present or absent), *p*=0.715.

### 3.2. Minnesota Satisfaction Questionnaire Items and Job Satisfaction

Inter-item reliability (Cronbach's alpha) of the short form of MSQ for our study was 0.767, indicating an acceptable reliability. Our nurse anesthetists were satisfied in ≥50.0% of cases in independence, variety, supervision—technical, moral value, security, and social service, authority, ability utilization, responsibility, creativity, and achievement. In more than seventy percent of cases, they were not satisfied with compensation, advancement, and working condition. Our nurse anesthetists were more satisfied with extrinsic MSQ scale than intrinsic scale, 53.5% vs. 44.3%, *p* = 0.001 (Table 2).

Our female nurse anesthetists were satisfied in >50.0% of the cases in variety, moral value, security, social service, authority, ability utilization, responsibility, creativity, co-workers, and achievements, while they were not satisfied to the same degree in activity, independence, compensation, advancement, and working conditions. More than 50.0% of our male nurse anesthetists were satisfied with independence, variety, social status, supervision—human relation, supervision—technical, moral value, security, social service, authority, ability utilization, responsibility, creativity, co-workers, recognition, and achievement, while more than 65.0% were not satisfied in compensation, advancement, and working conditions. Significantly more males were satisfied than females in independence, *p* = 0.011, supervision—human relation, *p* = 0.008, supervision—technical, *p* = 0.047, and recognition, *p* = 0.042. More females were not satisfied than males in compensation, *p* = 0.038, and creativity, *p* = 0.004. In the remaining MSQ items, non-satisfied vs. neutral, non-satisfied vs. satisfied, and neutral vs. satisfied, there was no statistically significant difference (Table 3).

### 3.3. Nurse Anesthetists' Job Satisfaction in Public and Private Hospitals

There was no significant difference in job satisfaction among the two groups. None of the nurse anesthetists practicing in public hospitals were satisfied with compensation; 16.7% of the nurse anesthetists practicing in private health sector reported satisfaction with compensation, though it was not statistically significant, *p*=0.065. Though not statistically significant, nurse anesthetists practicing in private health sector were more satisfied in their working conditions, 25.0% vs. 11.1%, *p*=0.363. Nurse anesthetists in public hospitals were not satisfied in (>50.0%) compensation, advancement, and working conditions, while nurse anesthetists in private hospitals were not satisfied in company policy and practice, compensation, and working conditions in >50.0% of the cases.

### 3.4. Reasons to Stay on or Leave Their Job

The main reason for our nurse anesthetists to stay on their post was to serve the public, 68.2%, and the other reasons were position change in 21.2%, further education in 24.2%, family related in 21.2%, and personal reasons in 13.6%. The main reasonsfor the nurse anesthetists to leave their job were family-related, personal reason, and change of profession in 6.1%, 15, 2%, and 15.2% of the cases, respectively. Non-satisfied nurse anesthetists had a significantly higher job-leaving response than satisfied nurse anesthetists, 74.4% vs. 36.8%, *p* = 0.016. Change of their position in their profession, further education, family reasons, or personal reasons were not statistically significantly associated with either to stay on or leave their job.

## 4. Discussion

Like in any other profession, multiple factors affect nurse anesthetists' JS. These factors are generally classified as intrinsic and extrinsic. Intrinsic factors include individual achievements, accomplishment, and prestige, while extrinsic factors include elements of the work environment such as pay and benefits, working conditions, and resources [7, 9, 10, 13, 19, 27].

### 4.1. Demographics

The response rate to our nurse anesthetists JS survey was 86.9%; this is comparable with similar studies that range from 83.6%–98.3% [2, 24, 28]. Gender distribution in our study was even, 50%; some reported male dominance that ranged from 52.4%–74.0% [2, 25, 28], while others reported female dominance that ranged from 53.2%–94.1% [1, 5, 7, 23, 27]. The marriage rate, 66.7%, in our study was in the reported range of 58.2%–85.5% [27, 28].

### 4.2. Gender and Job Satisfaction

The JS reports on gender are not uniform: absence of significant difference [7, 27, 29], significant satisfaction among females [5, 9, 10, 23], significant non-satisfaction among females [17, 27], and significant non-satisfaction among males [2]. We found significantly higher JS among our male nurse anesthetists, 57.6%. Factors that did not satisfy females the most were supervision, moral value, authority, creativity, and compensation [6]; in contrast, most of our female nurse anesthetists were neutral on supervision, but they were more satisfied with moral value, authority, and creativity and dissatisfied with compensation.

### 4.3. Age and Job Satisfaction

Some researchers found that the age group younger than 40 years constitutes the majority, 53.4%–84.7% [1, 27, 28]. Most of our nurse anesthetists were older than 40 years, 68.2%, that is consistent with some researchers who reported 56.5%–86.8% [17, 23, 29, 30] in the same age group. We did not find significant difference in JS among these two age groups, while many researchers [4, 9, 22, 23, 28, 31] found more JS in the age group ≥40 years. Pillay [23] in South Africa reported higher job satisfaction in >40 years of age group, especially in relationship with management and doctors. Kibwana et al. [13] found significant job satisfaction in 31–40 years age group, while Kinzl et al. in Switzerland found the contrary [29].

### 4.4. Marital Status and Job Satisfaction

The marriage rate in our study was 66.7%, and this agrees with similar studies that range from 64.0%–84.5% [24, 28–31], but others reported low marriage rates, 39.3%–46.9% [2, 7, 22, 25]. In our study, married nurse anesthetists had significantly higher JS than singles, unlike other reports which reported no significant difference [2, 7, 13] or non-satisfaction in the married group [27].

### 4.5. Educational Level and Job Satisfaction

The majority of our nurse anesthetists, 92.4%, were diploma holders; this is higher than the reported range, 54.0%–58.8% [7, 24, 27]. Conversely, our degree holders, 7.6%, were far fewer than the 39.0%–71.6% reported for the same credential [7, 25, 27]. We did not find significant JS difference among educational levels which is similar to the report by Abadiga et al. [7], but Admasu et al. [27] found significant non-satisfaction among degree holders. Our nurse anesthetists working in non-referral hospitals had significantly higher JS than those working in referral hospitals, while others reported more satisfaction in those nurse anesthetists working in higher teaching hospitals [27]. This may be partly due to extremely high workloads at our referral hospitals.

### 4.6. Length of Working Hours and Job Satisfaction

Most of our nurse anesthetists were working for more than 40 hours per week, and this is in agreement with reports from Iran and China [30, 31]. Our nurse anesthetists with ≥10 years' experience had significantly higher JS rate than those who worked for fewer years, and this is similar to the reports from Ethiopia and South Africa [9–11, 13, 23, 24] but is in contrast to another study from Ethiopia that did not find significant difference [7]. Similar to other studies [5, 23], our nurse anesthetists working in private hospitals were more satisfied with their job than those in public hospitals though it did not reach statistical significance.

### 4.7. Minnesota Satisfaction Questionnaire Items and Job Satisfaction

Increased JS is more influenced by the intrinsic factors than extrinsic factors [10], and this is in agreement with our significantly lower intrinsic MSQ items satisfaction and lower overall JS, 36.4%. Our nurse anesthetists' overall satisfaction rate is less than 41.4%–60.8% which is reported by others in LMICs [2, 5, 9, 10, 23, 27, 28]. More than 50.0% of our nurse anesthetists were satisfied in their independence, variety, supervision—technical, moral value, security, social service, autonomy, authority, responsibility, creativity, and achievement, but they were not satisfied in compensation, advancement, and achievement. Similarly, many researchers reported non-satisfaction in relation to compensation and advancement [1, 2, 9, 11, 13, 27, 29, 30]. Mousavi et al. from Iran did not finddissatisfaction in advancement[30]. Rukewe et al. reported an absence of significant effect of compensation and advancement on JS [28]. Similar to our findings, some researchers reported higher satisfaction with independence [5, 22, 27], social service [2, 5, 9, 22, 27], advancement [22], recognition [2, 27], and responsibility [5, 22, 30]. Our JS level findings in regard to working condition, 13.6%, and recognition, 37.9%, were far lower than 43.9% and 49.0% reported, respectively, by Fentie et al. [22]. Our nurse anesthetists' achievement, 60.0%, was higher than that reported by Desalegn et al., 33.3% [5]. We did not find significant association between JS and activity and being busy at work, which is similar to the report by Kinzl et al. from Switzerland [29]. Kinzl et al. [29] reported absence of significant association between JS and variety of work, while Mahoney et al. from the United States [4] reported skill variety and autonomy result in higher JS. We found that 80.3% of the nurse anesthetists were not satisfied with their pay which is higher than 36.5% reported by Gebregziabher et al. [25].

### 4.8. Job Satisfaction in Public vs. Private Hospitals

Our nurse anesthetists in public hospitals were not satisfied in compensation, advancement, and working conditions in more than 50.0% of the cases. The overall poor job satisfaction in our nurse anesthetists working in public hospitals was similarly reported by others [9, 23]. The common sources of satisfaction for public hospital nurse anesthetists emanate from the social context of the work [9, 23]; this is consistent with our findings. Private nurses reported satisfaction in patient care, relationship with nurses and doctors, and work environment but registered non-satisfaction in their pay and carrier development opportunities [9, 19]. Our private sector nurse anesthetists were satisfied in social service, autonomy, and ability utilization, but they were not satisfied in supervision—human relation, company policy and practices, compensation, advancement, and working conditions.

### 4.9. Factors Involved in Leaving or Staying on Their Job

Globally, anesthetists leave their jobs due to stress, fatigue, and long working hours [22]. The most important factor to keep our nurse anesthetists in their job was serving the public. Our study shows that 28.8% of our nurse anesthetists stay in their job because of their JS in contrast to 13.7% reported by Desalegn et al. [5]. Further education is reported in 24.2% of our nurse anesthetists as a reason to remain in their job, while others reported 20.0% of their nurse anesthetists stayed for educational opportunities [5]. Kols et al. found absence of professional development as an important factor in nurse anesthetists quitting their job [11]. Generally non-satisfied nurse anesthetists quit their job [4]. In our study, non-satisfaction in the job accounted for 66.7% in the reason to quit their job; others reported a lower rate of 30.6%–57.0% [2, 5]. Our nurse anesthetists may leave their job in 6.1% of cases for family-related issues; this is lower than 16.5% reported by Mahoney et al. from the United States [4]. Personal-related issues were described by 15.2% of our nurse anesthetists as a reason to leave their job, and this is lower than the 27.8% reported by Yami et al. [2]. Only 15.2% of our nurse anesthetists leave their job to change their profession while others reported higher, 32.6% [5]. Similar to the report by Kols et al. [11], gender was not an important factor to stay on or quit the job.

### 4.10. Limitations and Strength

The cross-sectional and self-administered nature of the study may not demonstrate a causal relationship between variables. Although the Minnesota Satisfaction Questionnaire is a validated tool to evaluate the JS, it is not specifically designed for nurse anesthetists. The number of nurse anesthetists from the private sector was few, 12. A response rate of 86.9% of all nurse anesthetists currently in clinical practice in Botswana and involving all levels and types of hospitals are the strengths of this study.

## 5. Conclusions

This study demonstrated the low JS of nurse anesthetists currently practicing in Botswana in both public and private hospitals. Most nurse anesthetists were satisfied with independence, variety, supervision—technical, moral value, security, social service, authority, ability utilization, responsibility, and creativity, but they were not satisfied in compensation, working conditions, and advancement. The health managers in Botswana need to develop strategies to improve the low JS level among nurse anesthetists currently in practice in the country. Many studies [5, 6, 22] demonstrated that a positive JS translates to a longer stay on their job as well as improved patients' satisfaction and their care. Some of the determinants of JS identified in this study may provide an important input in addressing the low JS among our nurse anesthetists. Since nurse anesthetists work in multidisciplinary teams that involve other medical specialties, we recommend similar studies for related medical professions in Botswana.

## Figures and Tables

**Table 1:** The effect of gender, marital status, citizenship, hospital level, and years of experience on overall job satisfaction of nurse anesthetists in Botswana, 2020.

Factors		Satisfied	Not satisfied	*p* value
Gender	Female	5 (15.2%)	28 (84.8%)	**0.001**
	Male	19 (57.6%)	14 (42.4%)	

Marital status	Single	4 (19.0%)	17(81.0%)	**0.039**
	Married	20 (45.5%)	24 (54.5%)	

Citizenship	Local	11 (23.4%)	36(76.6%)	**0.001**
	Expat	13 (68.4%)	6 (31.6%)	

Hospital level	Referral	1 (7.7%)	12 (92.3%)	**0.023**
	Non-referral	23 (43.4%)	30 (56.6%)	

Experience in years	<10	4 (17.4%)	19 (82.6%)	**0.019**
	≥10	20 (46.5%)	23 (53.5%)	

**Table 2:** Short form of Minnesota Satisfaction Questionnaire and level of job satisfaction among nurse anesthetists in Botswana, 2020.

MSQ items	Satisfied	Neutral	Not satisfied
Being able to keep busy all the time (activity)^*∗*^	23 (34.8%)	13 (19.7%)	29 (43.9%)
The chance to work alone on the job (independence)^*∗*^	**33 (50.0%)**	10 (15.2%)	23 (34.8%)
The chance to do different things from time to time (variety)^§^	**37 (56.1%)**	18 (27.3)	11 (16.7%)
The chance to be “somebody” in the community (social status)^*∗*^	29 (43.9%)	19 (28.8%)	17 (25.8%)
The way my boss handles his/her workers (supervision—human relation)	26 (39.4%)	25 (37.9%)	15 (22.7%)
The competence of my supervisor in making decisions (supervision—technical)	**35 (53.0%)**	20 (30.3%)	11 (16.7%)
Being able to do things that do not go against my conscience (moral value)^*∗*^	**35 (53.0%)**	9 (13.6%)	19 (28.8%)
The way my job provides for steady employment (security)^§^	**49 (74.2%)**	12 (18.2%)	5 (7.6%)
The chance to do things for other people (social service)^*∗*^	**52 (78.8%)**	11 (16.7%)	3 (4.5%)
The chance to tell people what to do (authority)^§^	**40 (60.6%)**	20 (30.3%)	6 (9.1%)
The chance to do something that makes use of my abilities (ability utilization)^*∗*^	**46 (69.7%)**	12 (18.2%)	8 (12.1%)
The way company policies are put into practice (company policy and practices)^§^	17 (25.8%)	19 (28.8%)	29 (43.9%)
My pay and the amount of work I do (compensation)^*∗*^	2 (3.0%)	10 (15.2%)	**53 (80.3%)**
The chances for advancement on this job (advancement)^*∗*^	6 (9.1%)	7 (10.6%)	**52 (78.8%)**
The freedom to use my own judgment (responsibility)^§^	**44 (66.7%)**	16 (24.2%)	6 (9.1%)
The chance to try my own methods of doing the job (creativity)^*∗*^	**37 (56.1%)**	19 (28.8%)	9 (13.6%)
The working conditions (working condition)^*∗*^	9 (13.6%)	9 (13.6%)	**48 (72.7%)**
The way my co-workers get along with each other (co-workers)^*∗*^	**39 (59.1%)**	15 (22.7%)	12 (18.2%)
The praise I get for doing a good job (recognition)^§^	25 (37.9%)	20 (30.3%)	20 (30.3%)
The feeling of accomplishment I get from the job (achievement)^*∗*^	**40 (60.6%)**	16 (24.2%)	10 (15.2%)

^*∗*^Intrinsic scale; ^§^extrinsic scale.

**Table 3:** Short form of Minnesota Satisfaction Questionnaire, gender, and significant level of nurse anesthetists' job satisfaction in Botswana, 2020.

MSQ items		Not satisfied (%)	Neutral (%)	Satisfied (%)	*p* value
The chance to work alone on the job (independence)	Female	56.7	—	43.3	0.011
	Male	23.1	—	76.9	

The chance to work alone on the job (independence)	Female	85.0	15	—	0.026
	Male	46.2	53.8	—	

The way my boss handles his/her workers (supervision—human relation)	Female	—	68.0	32.0	0.008
	Male	—	30.8	69.2	

The competence of my supervisor in making decisions (supervision—technical)	Female	—	50.0	50.0	0.047
	Male	—	24.1	62.9	

My pay and the amount of work I do (compensation)	Female	66.7	33.3	—	0.038
	Male	26.7	73.3	—	

The chance to try my own methods of doing the job (creativity)	Female	61.5	38.5	—	0.004
	Male	6.7	93.3	—	

The praise I get for doing a good job (recognition)	Female	—	63.6	36.4	0.011
	Male	—	26.1	73.9	

## Data Availability

The data used to support the findings of this study are available from the corresponding author upon request.
